# Statins and 90-Day Functional Performance and Survival in Patients with Spontaneous Intracerebral Hemorrhage

**DOI:** 10.3390/jcm12206608

**Published:** 2023-10-19

**Authors:** Karolina Zaryczańska, Wioletta Pawlukowska, Przemysław Nowacki, Łukasz Zwarzany, Ewelina Bagińska, Monika Kot, Marta Masztalewicz

**Affiliations:** 1 Department of Neurology, Pomeranian Medical University, 71-252 Szczecin, Poland; wioletta.pawlukowska@pum.edu.pl (W.P.); przemyslaw.nowacki@pum.edu.pl (P.N.); marta.masztalewicz@pum.edu.pl (M.M.); 2 Department of Diagnostic Imaging and Interventional Radiology, Pomeranian Medical University, 71-252 Szczecin, Poland; lukasz.zwarzany@pum.edu.pl; 3 Independent Researcher, 71-004 Szczecin, Poland; monikakot26@gmail.com

**Keywords:** stroke, spontaneous intracerebral hemorrhage, sICH, statins, functional capacity

## Abstract

Background: The neuroprotective effect of statins has become a focus of interest in spontaneous intracerebral hemorrhage (sICH). The purpose of this study was: (1) to evaluate the effect of statin use by the analyzed patients with sICH in the period preceding the onset of hemorrhage on their baseline neurological status and baseline neuroimaging of the head; (2) to evaluate the effect of statin use in the acute period of hemorrhage on the course and prognosis in the in-hospital period, taking into account whether the statin was taken before the hemorrhage or only after its onset; (3) to evaluate the effect of continuing statin treatment after in-hospital treatment on the functional performance and survival of patients up to 90 days after the onset of sICH symptoms, taking into account whether the statin was taken before the onset of sICH. Material and Methods: A total of 153 patients diagnosed with sICH were analyzed, where group I were not previously taking a statin and group II were taking a statin before sICH onset. After lipidogram assessment, group I was divided into patients without dyslipidemia and without statin treatment (Ia) and patients with dyslipidemia who received de novo statin treatment during hospitalization (Ib). Group II patients continued taking statin therapy. We evaluated the effect of prior statin use on the severity of hemorrhage; the effect of statin use during the acute period of sICH on its in-hospital course; and the effect of statin treatment on the severity of neurological deficit, functional capacity and survival of patients up to 90 days after the onset of sICH symptoms. Results: There was no effect of prior statin use on the severity of hemorrhage as assessed clinically and by neuroimaging of the head. At in-hospital follow-up, subgroup Ia was the least favorable in terms of National Institutes of Health Stroke Scale (NIHSS) score. This subgroup had the highest percentage of deaths during hospitalization. In the post-hospital period, the greatest number of patients with improvement in the NIHSS, modified Rankin Scale (mRS) and Barthel scales were among those taking statins, especially group II patients. At 90-day follow-up, survival analysis fell significantly in favor of subgroup Ib and group II. Conclusions: 1. The use of statins in the pre-sICH period did not adversely affect the patients’ baseline neurological status or the results of baseline neuroimaging studies. 2. Continued statin therapy prior to the onset of sICH or the inclusion of statins in acute treatment in patients with sICH and dyslipidemia does not worsen the course of the disease and the in-hospital prognosis. Statin therapy should not be discontinued during the acute phase of sICH. 3. To conclude the eventual beneficial effect on the functional performance and survival of patients after sICH onset, comparability of the analyzed groups in terms of clinical, radiological and other prognostic factors in spontaneous intracerebral hemorrhage would be needed. Future studies are needed to confirm these findings.

## 1. Introduction

Statins, by their hypolipidemic effect, as well as immunomodulating, anti-inflammatory, and antiaggregation effects, play an important role in stabilizing atherosclerotic lesions [1,2,3]. These properties give them an important place in the prevention of acute coronary syndromes and atherosclerotic acute cerebral ischemic episodes [4,5,6].

Experimental studies have shown that statins, in addition to their protective effect against ischemic stroke, also play a role in reducing brain damage in acute ischemic conditions [7,8]. The beneficial effects of statins on ischemic stroke have also been found in both observational and randomized studies [9].

At the same time, there is a debate whether statins, as cholesterol-lowering drugs (the building material of the cell wall) and also having an antiaggregation effect, increase the risk of intracerebral hemorrhage. In addition to studies on the possible negative effect of statins on the risk of sICH [10,11], there are papers supporting neutral or even beneficial, protective, effects of statins on sICH [12,13,14].

The potential beneficial effects of statins lie in their neuroprotective effects in the border of neural tissue and hematoma. They are supposed to contribute to the reduction in edema and inflammation in the zone of blood–brain barrier damage through their antiapoptotic, anti-inflammatory, antioxidant and angiogenesis-stimulating effects [15,16,17,18,19,20,21,22].

Studies indicating the negative effects of statins suggest that low cholesterol levels may impair endothelial integrity and affect the susceptibility of the arterial wall to extravasation [15]. Statins’ inhibition of platelet activity and the coagulation cascade may promote an increase in hematoma size [23].

Another topic is the intake of statins by patients who have suffered intracerebral hemorrhage. It is recommended not to discontinue previous statin treatment to avoid the “rebound” effect after their withdrawal. It may result in oxidative stress, impaired vascular function and adverse clinical outcomes [24,25]. There is caution about the inclusion of de novo statins in patients with sICH, although there are a growing number of studies showing significant benefits from such treatment [25]. Nevertheless, there are no definitive guidelines so far from the most prestigious societies, including the American Heart Association (AHA) and the American Stroke Association (ASA) as to the treatment with statins in acute sICH [26].

The use of statins in patients with intracerebral hemorrhage would be supported by the fact that these patients tend to have atherosclerotic risk factors for stroke and are at high risk for developing a disabling ischemic stroke [27]. In addition, experimental studies imply a neuroprotective effect of statins in the area of intracerebral hemorrhage and a beneficial effect on recovery from hemorrhage [28,29]. The results of analyses in humans seem to confirm this observation. However, most of these are observational, retrospective studies [30,31,32,33]. Prospective studies may provide stronger evidence for the use of statins in this population.

The objective of this study was to assess whether or how the use of statins prior to sICH affects the initial neurological status and the result of the primary radiological examination. In addition, the objective was to prospectively evaluate whether continuing statin treatment prior to spontaneous intracerebral hemorrhage or including statins in the treatment process during the acute period worsens its course and in-hospital prognosis. Finally, the aim of this study was to assess whether continuing statin treatment after in-hospital treatment affects the functional performance and survival of patients up to 90 days after the onset of spontaneous intracerebral hemorrhage symptoms.

## 2. Material and Methods

### 2.1. Materials

Consecutive patients over the age of 18 with a diagnosis of spontaneous intracerebral hemorrhage hospitalized at the Department of Neurology with Stroke Unit of the Pomeranian Medical University in Szczecin between March 2017 and December 2020 were included in this study.

Exclusion criteria were: (1) confirmed secondary bleeding into the central nervous system (e.g., tumor, vascular malformation, hemorrhagic transformation after ischemic stroke, coagulopathy, and anticoagulation treatment), (2) subarachnoid hemorrhage, (3) intracranial post-traumatic hemorrhage, (4) accompanying recent cerebral ischemic foci, (5) fever and/or high inflammatory markers on the day of admission to the hospital, (6) contraindications to statin treatment based on the characteristics of the drug product, i.e., liver disease, elevated transaminase activity three times above the upper limit of normal, kidney disease, thyroid disease, muscle disease, use of cyclosporine, fibrates, and drug hypersensitivity.

A total of 153 patients were enrolled in this study, including 70 women and 83 men aged 36 to 95 years.

### 2.2. Methods

#### 2.2.1. Qualification for This Study

##### Recognition of sICH

The confirmation of sICH was based on a neurological examination (history, physical examination with assessment of neurological status), the result of a neuroimaging study—computed tomography (CT) or head magnetic resonance imaging (MRI); in selected cases, it was expanded to include a vascular angiography option, and on the basis of the results of laboratory tests that allowed us to exclude secondary causes of bleeding.

Relevant to this study from the history were: (1) age; (2) cigarettes, alcohol; (3) past use of statins, consideration of possible contraindications to their use; (4) past use of antiaggregants, anticoagulants; (5) comorbidities—hypertension, type 2 diabetes mellitus, atrial fibrillation, dyslipidemia, ischemic heart disease; (6) history of myocardial infarction, history of ischemic stroke, history of intracerebral hemorrhage; (7) circumstances of current symptoms.

The physical examination, in addition to an internal medicine evaluation (with assessment of temperature, Body Mass Index (BMI), blood pressure, and signs of hemorrhagic diathesis), included an assessment of the severity of the neurological deficit associated with sICH, based on the NIHSS [34].

The neuroimaging examinations were mostly performed on a CT scanner with a 32-row detector, 64 layers in acquisition, a minimum gantry rotation time of 0.33 s and a minimum layer thickness of 0.6 mm. Other examinations were performed on a CT scanner with a 64-row detector, 128 layers in acquisition, a minimum gantry rotation time of 0.28 and a minimum layer thickness of 0.5 mm.

In selected cases, neuroimaging diagnostics was extended to magnetic resonance imaging. For this purpose, a 1.5 tesla scanner was used, with gradients of 50 mT (millitesla/meter) in all three axes. The angio-MRI option was enabled by an HNS HEAD (head-neck-spine) multichannel coil for head and neck imaging, with 18 transmit/receive channels.

The head neuroimaging data analyzed were:

– Size (volume) of the intracerebral hematoma in mm^3^ (according to the ABC/2 rule, where A is the anterior–posterior dimension, B right-left, C inferior-posterior) [35].
– Presence of cerebral edema,
– Penetration into the ventricular system, and
– Displacement of intracranial structures.

Among the laboratory tests performed on admission, the results of morphology, blood glucose, coagulation system, C-Reactive Protein (CRP) and, at a later stage of this study, lipidogram determined up to the 3rd day of hospitalization were important for qualification.

Patients with sICH and coexisting newly diagnosed hypertension, diabetes, atrial fibrillation and dyslipidemia during hospitalization were also included in this study.

(1) Hypertension was diagnosed according to the 2018 Guidelines of the European Society of Cardiology (ESC) and the European Society of Hypertension (ESH), in which hypertension was defined as a reported systolic blood pressure (SBP) ≥ 140 mm Hg and/or diastolic blood pressure (DBP) ≥ 90 mm Hg based on clinical/physician office measurements [36].
(2) Diabetes mellitus was diagnosed based on the 2018 Polish Diabetes Association criteria after excluding transient hyperglycemia due to stroke [37].
(3) Atrial fibrillation was diagnosed based on a 12-lead ECG (electrocardiogram) following the 2016 ESC guidelines [38].
(4) Dyslipidemia was defined as abnormal plasma concentrations of either lipid fractions or lipoproteins. The term includes any abnormality in the lipidogram regarding total cholesterol (TC), high-density lipoprotein (HDL), low-density lipoprotein (LDL) and triglycerides (TG) fractions [39]. This study used the standards of the Polish Society of Cardiology (PTK) and the Polish Society of Laboratory Diagnostics (PTDL), which also apply to the hospital laboratory. The normal values at the start of this study were TC 115.00–190.00 mg/dL, HDL ≥ 45 mg/dL, and LDL < 115 mg/dL in healthy subjects and those with low or moderate cardiovascular risk), <100 mg/dL for patients at high risk and <70 mg/dL with very high risk of cardiovascular events, and TG ≤ 150 mg/dL [40]. For the purpose of this study, we assumed an LDL fraction concentration >115 mg/dL qualifying for statin inclusion.

Approval was obtained from the Bioethics Committee of the Pomeranian Medical University dated 16 January 2017 no. KB-0012/18/17.

##### Group Assignment

Consecutive patients diagnosed with sICH were assigned to one of two groups.

Group I included patients who were not taking a statin before they developed sICH. Group II included patients previously taking a statin (atorvastatin or rosuvastatin) for at least 6 months before the onset of sICH.

Eligibility for the mentioned groups was based in the first stage (stage I) on a history of taking statins before the disease. In patients previously taking statins (group II), the name of the drug and the dose were important.

Then, in the second stage (stage II), group I was divided into subgroups Ia and Ib, based on the exclusion or confirmation of dyslipidemia in them, based on the determination of lipidogram (TCH, LDL, HDL, TGR) no later than the 3rd day of hospitalization.

Subgroup Ia patients, without dyslipidemia, were not taking statins before the onset of sICH, and in them, according to the study design, no such treatment was administered after the onset of sICH.

In subgroup Ib, statins were started in patients with dyslipidemia during the acute period of sICH with a recommendation to continue at least until day 90, including in-hospital and out-of-hospital periods.

Group II, patients taking statins before developing sICH, continued taking the drug in the acute period of the disease with the recommendation of continuation for at least 90 days, including the in-hospital and post-hospital periods.

Initially, the size of each subgroup and group II was expected to be at least 30 patients.

#### 2.2.2. Course of the Study (Figure 1)

##### Stage 1

Patients meeting the eligibility criteria were assigned to group I (not previously taking statins) and group II (taking statins). This stage of the study involved comparing group I with group II on admission in terms of selected clinical-demographic data obtained sequentially from history, physical examination, neuroimaging and laboratory tests.

##### Stage 2

After the lipidogram was determined by the 3rd day of hospitalization and dyslipidemia was ruled out or confirmed, group I was divided into the previously mentioned subgroups Ia and Ib. Those who were not taking statins and who were not diagnosed with dyslipidemia (subgroup Ia) did not receive statins. Patients with newly diagnosed dyslipidemia (subgroup Ib) received atorvastatin at a dose of 20 mg/day, with a recommendation to take it at least until the 90th day after the onset of sICH.

In group II patients, continuation of a previously used statin (atorvastatin or rosuvastatin) was maintained at previously taken doses, if they were low at the start of this study, doses were increased for atorvastatin to 20 mg/d and rosuvastatin to 10 mg/d.

Considering the studies in which the authors indicate an increased risk of hemorrhage with the use of high doses of statins for the course of sICH we focused on low doses of statins [41].

The evaluation of selected clinical-demographic data, neurological status on the NIHSS scale and assessment of radiological changes was repeated as in stage I, with division into subgroups Ia, Ib and group II. Additional assessments were made of neurological status on the NIHSS scale, functional capacity on the mRS [42] and Barthel [43] scales on the day of hospital discharge.

The effect of statins in the acute period of sICH on its in-hospital course was analyzed.

##### Stage 3

Subgroups Ia, Ib and group II were analyzed in terms of neurological deficit, functional performance on the mRS and Barthel scales 90 days after the onset of sICH.

Follow-up of neurological status and assessment of functional performance 90 days after the onset of sICH was carried out by direct examination of the patient or by telephone interview with the patient or caregiver based on a standardized set of questions, according to previously used scales (NIHSS, Barthel and mRS). Based on these scales, the neurological deficit and the degree of the patient’s independence in activities of daily living were determined, comparing the results with the scores from the day of hospital discharge.

Post-hospital mortality of patients was also taken into account at this stage.

##### Stage 4

It included an analysis of patient survival.

**Figure 1:**
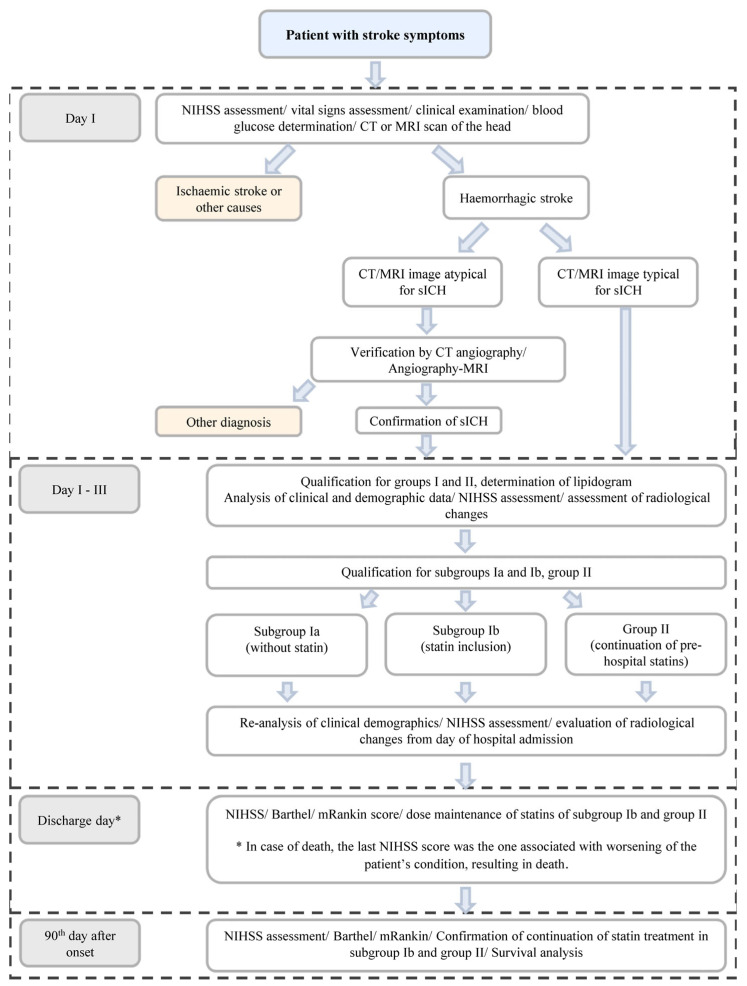
Schematic of the conducted study. * In the case of death (assumed as the discharge day), the last NIHSS score was the one associated with worsening of the patient’s condition, resulting in death.

### 2.3. Statistical Analysis

Arithmetic means, minimum and maximum values, standard deviations, medians, lower and upper quartiles were used to describe numerical variables. To represent qualitative data, the percentage and abundance of the variable were given. Levene’s test showed heterogeneity of variance in the study groups and patient subgroups (*p* < 0.05). For this reason, and because the distribution of variables deviated from a normal distribution (Shapiro–Wilk test, *p* < 0.05), non-parametric tests were used to compare groups of variables: for two groups, the Mann–Whitney U test; for three groups, the Kruskal–Wallis rank ANOVA test and the median test. Dependent variables were compared using the Wilcoxon paired rank-order test. Pearson’s chi-square test for 2 × 2 tables was used to compare qualitative variables. Survival differences between groups were illustrated using Kaplan–Meier curves. In addition, the study variables were presented using box plots or histograms. Statistical significance was assumed at *p* < 0.05. The analysis was performed using Statistica version 13.3 (StatSoft Inc., Krakow, Poland) under a current license.

## 3. Results

### 3.1. Analysis of Patients Not Using Statins in the Period before the Onset of sICH (Group I) and Patients Using Statins before the Onset of sICH (Group II)

#### 3.1.1. Intake of Statins before SICH in Relation to Age, Gender, Vascular Risk Factors for Stroke and Current Lipid Profile

Patients taking statins before the onset of sICH (group II) were significantly older than patients who had not taken the drug before (group I). They were significantly more likely to have dyslipidemia, ischemic heart disease, type 2 diabetes. They were significantly more likely to have had myocardial infarction, ischemic stroke. Additionally, they were significantly more likely to take antiaggregant drugs (Table 1).

The groups were comparable in gender distribution, BMI values, smoking and alcohol consumption, previously diagnosed hypertension, atrial fibrillation (Table 1).

However, the fact of previous intracerebral hemorrhage in the group of patients who had previously taken statins was reported less frequently than among patients who had not previously taken statins. This difference was not statistically significant (Table 1).

At the time of hospital admission, patients in group II had significantly higher glycemic values than those in group I. Median SBP and DBP values were comparable in both groups. In the lipidogram measured up to the 3rd day of hospitalization, group II patients had significantly lower values of total cholesterol fraction and LDL fraction than group I patients. The groups were comparable in levels of triglycerides (Table 1).

#### 3.1.2. Statin Intake before sICH and Severity of Hemorrhage in Clinical and Neuroimaging Evaluation

Group I and II patients did not differ significantly at the time of admission in the severity of the neurological deficit associated with intracerebral hemorrhage according to the NIHSS scale (Table 2).

There were no significant differences between them on neuroimaging assessments of hematoma volume, the presence of cerebral edema, the degree of displacement of midline structures, the presence of penetration of the hematoma into the ventricular system, and subarachnoid intussusception (Table 2).

### 3.2. Analysis of Patients Who Did Not Use Statins in the Period Prior to sICH and in the Acute Period of sICH (Group Ia), Patients Who Were Started on Statins in the Acute Period of sICH (Group Ib), and Patients Who Continued Statin Treatment Initiated Prior to the Onset of sICH (Group II)

#### 3.2.1. Statin Intake before sICH in Relation to Age, Gender, Vascular Risk Factors for Stroke and Current Lipid Profile

After the division of group I into subgroups Ia and Ib was considered, group II patients were significantly older than those of the mentioned subgroups, with no age difference between subgroups Ia and Ib (Table 3).

Group II patients were significantly more likely to have a diagnosis of ischemic heart disease compared to subgroups Ia and Ib, and were significantly more likely to take antiaggregants. Subgroups Ia and Ib did not differ in this regard (Table 3).

There were no significant differences between group II and subgroups Ia and Ib with regard to previous history of myocardial infarction, ischemic stroke, although most such patients were in group II (Table 3).

Type 2 diabetes was most common in group II, followed by subgroup Ia. The least frequent in subgroup Ib. However, these differences were not statistically significant (Table 3).

Group II, subgroup Ia and Ib were comparable in gender and BMI distribution, smoking, alcohol consumption, prevalence of hypertension, atrial fibrillation (Table 3). The percentage of patients with a prior history of intracerebral hemorrhage was highest in subgroup Ia, but without statistical significance (Table 3).

Baseline SBP and DBP values were comparable in both subgroups and group II (Table 3).

Subgroup Ib patients compared to subgroup Ia and group II had significantly higher values of total cholesterol and LDL fraction. Patients in subgroup Ia and group II did not differ significantly in these respects. There were no significant differences in HDL cholesterol and triglyceride fractions between the subgroups and group II (Table 3).

Glycemia on admission was similar between group II and subgroup Ia and higher than in subgroup Ib, although without statistical significance (Table 3).

#### 3.2.2. Statin Intake before sICH versus Hemorrhage Severity in Clinical and Neuroimaging Evaluation

The NIHSS neurological deficit at admission was significantly greater in subgroup Ia compared to subgroup Ib and comparable to group II. There was no significant difference in deficit at baseline between subgroup Ib and group II patients (Table 4).

Hemorrhagic foci volume was significantly larger in subgroup Ia compared to subgroup Ib, but with no significant difference from group II. There was also no significant difference between subgroup Ib and II in this regard (Table 4).

Midline shift was significantly greater in patients of subgroup Ia relative to subgroup Ib, but with no significant difference relative to group II. There was also no significant difference between subgroup Ib and group II (Table 4).

Subepithelial intussusception was found significantly more often in patients in subgroup Ia relative to subgroup Ib, with no significant differences in the other combinations (Table 4).

The penetration of the sICH into the ventricular system was significantly more frequent in subgroup Ia relative to subgroup Ib and group II. Subgroup Ib and group II did not differ (Table 4).

There was a comparable percentage of patients with cerebral edema in the subgroups and group II (Table 4).

#### 3.2.3. Statin Intake before and after Spontaneous Intracerebral Hemorrhage and the Clinical Course of Hemorrhage in Analyzed Patients in the In-Hospital Period

At in-hospital follow-up, the percentage of patients with improvement in neurological deficit on the NIHSS scale on the day of discharge in subgroup Ib and group II was significantly higher than in subgroup Ia. In subgroup Ib and group II, the percentage was similar (Table 4).

In the in-hospital period, the highest percentage of deaths was observed in subgroup Ia and was significantly higher relative to subgroup Ib. There was no significant difference in mortality rates between the aforementioned subgroups and group II (Table 4).

#### 3.2.4. Statin Intake in the Period before and after Spontaneous Intracerebral Hemorrhage vs. Neurological Status, Functional Capacity and Survival in Analyzed Patients within 90 Days after the Onset of sICH

In post-hospital follow-up up to day 90, further improvement in the NIHSS scale was noted in all groups analyzed (Ia, Ib and II) compared to the study on the day of discharge. The percentage was highest in group II, second in subgroup Ib; lowest in subgroup Ia. The difference was found to be statistically significant between subgroups Ib and Ia, and group II and subgroup Ia. The percentage of patients with improvement in subgroup Ib and group II was statistically comparable (Table 4).

The degree of disability according to the mRS and Barthel scale on the day of discharge was highest in subgroup Ia, and lowest in subgroup Ib. A statistically significant difference was found between the two subgroups. The score obtained by group II patients was statistically comparable to subgroups Ia and Ib (Table 4).

Analysis of the mRS and Barthel scale scores after 90 days showed further improvement in functional performance in all analyzed groups (Ia, Ib, II). The percentage of patients with improvement from the day of discharge in the mentioned scales was significantly higher in subgroup Ib and group II compared to subgroup Ia, while subgroup Ib and group II did not differ significantly (Table 4).

Significantly fewer patients died during the post-hospital period compared to the hospitalization period, with the lowest number in group II. However, the differences in the percentage of deaths between the groups were not significant (Table 4).

A total of 29.4% of deaths (45 patients) in the study population were recorded between hospital admission and 90 days after the illness.

At 90-day follow-up, survival analysis fell significantly in favor of subgroup Ib and group II (those receiving statins). Most deaths occurred up to the 10th day of illness (group Ia and II).

## 4. Discussion

Treatment options for patients with spontaneous intracerebral hemorrhage are limited due to the lack of specific therapy. Therapeutic management includes control of hypertension, treatment of cerebral edema, physical and speech rehabilitation, and measures to prevent complications in patients who are lying down and in serious condition [44]. Extravasation of blood into the brain results in a cascade of reactions leading to cell damage in the hemorrhagic area, with possible expansion of hemorrhagic lesions, development of cerebral edema. The area of the brain eventually damaged depends on the evolution of these lesions [45]. The possibility of additional therapies to protect the brain from the unfavorable evolution of lesions in the hemorrhagic area could improve the prognosis of patients with sICH.

Experimental studies suggest a neuroprotective effect of statins in the area of intracerebral hemorrhage and their beneficial effect on recovery from hemorrhage [28,29]. Previous studies in patients with sICH seem to confirm this. However, they are mostly observational, retrospective studies [30,31,32,33]. Our study was a prospective study.

In the first stage, the association between the use of statins prior to sICH with the severity of hemorrhage was analyzed. For that, patients with sICH who had taken statins before becoming ill were compared with patients with sICH who had not taken these medicines before. Patients taking statins before sICH (group II) were significantly older, with a higher baseline burden of cardiovascular risk factors compared to the other patients (group I). They were significantly more likely to take antiaggregants than the other patients. They had significantly higher, although not very high, blood glucose levels on admission. Regardless of these unfavorable factors [46], patients of group II did not differ from patients of group I in the assessment of the severity of the hemorrhage, considering the severity of the neurological deficit on the NIHSS scale on admission and the volume of the hemorrhagic foci, the penetration of the hematoma into the ventricular system, the accompanying hemorrhage and the mass effect on the initial neuroimaging study of the head.

According to the results, the use of statins by our patients prior to the onset of sICH did not adversely affect their initial neurological status and the results of initial neuroimaging examinations compared to patients without the therapy. In fact, the absence of the differences could be seen as a sign of the clinical benefit of previous use of statins by the more severely affected patients of group II.

The fact that group II patients had better results of lipidogram, LDL fraction and total cholesterol on admission than group I patients confirms that these patients are under the hypolipemic effect of statins.

In previous theories, although not abandoned in the end, the prevailing view was that lowering concentrations of cholesterol, as the building material of the cell wall, could lead to a weakening of the integrity of the vessel wall, thereby increasing its susceptibility to extravasation [15]. It has been suggested that hypocholesterolemia affects the increased risk of intracerebral hemorrhage at least in patients with high hypertension [24,47,48]. The theory of adverse effects of statins in studies was supported by statins’ inhibition of platelet activity and the coagulation cascade—which could indirectly suggest a predisposition to increase hematoma size [23].

In the presented study, the volume of the hemorrhagic foci and its accompanying consequences, as revealed by neuroimaging, were comparable in both groups. The findings of previous studies in patients with intracerebral hemorrhage, available in the literature, which have taken into account the association of statin intake prior to hemorrhage with the patients’ baseline clinical status and radiographic picture of the hemorrhage, also did not show an adverse effect of statins in this aspect [49], and some of them even suggest a better clinical status of these patients compared to patients not taking statins prior to hemorrhage [50].

In the following stages of this study, we analyzed the effect of statin intake by patients with intracerebral hemorrhage on the in-hospital course of the hemorrhage, and then on the functional capacity of patients with sICH 90 days after the onset. All group II patients had continued statin treatment after the onset of hemorrhage. Group I patients with hemorrhage who were diagnosed with dyslipidemia (subgroup Ib) based on a lipidogram determined no later than 3 days after the incident also received statins.

Group I patients without dyslipidemia (subgroup Ia) did not receive statins. Each group (group II and both subgroups I) showed improvement in the severity of hemorrhage-related symptoms during their hospital stay. It should be noted, however, that the percentage of patients with improvement in neurological deficits on the NIHSS scale on the day of discharge in patients taking statins was significantly higher than in patients not taking statins, with no difference in whether the drug was taken before the hemorrhage occurred or only after the hemorrhage occurred.

The percentage of deaths among patients not taking statins at all was significantly higher than in patients who started receiving them after the onset of hemorrhage, and comparable to patients who took them before the onset of hemorrhage and had continued treatment after the onset of hemorrhage.

Is it possible to say that in the patients we analyzed with sICH, taking statins had a beneficial effect on the course of hemorrhage in the early post-onset period?

Group II patients were significantly older than the patients of the mentioned subgroups, with no significant age difference between the subgroups. Older age could imply a worse prognosis regarding the course of hemorrhage, functional performance after hemorrhage [51,52,53,54], and in the case of our patients, despite their older age, group II performed similarly favorably to subgroup Ib. Group II patients were significantly more likely to have a diagnosis of ischemic heart disease compared to subgroups Ia and Ib, and were significantly more likely to take antiaggregants. Subgroups Ia and Ib did not differ in this regard. There were no significant differences between group II and subgroups Ia and Ib with regard to previous myocardial infarction, ischemic stroke, although the largest number of such patients was in group II. These factors could have projected a worse course of hemorrhage in group II patients [55,56,57], which we did not observe. This raises the idea that taking statins before the onset of hemorrhage measurably influenced the favorable in-hospital course of hemorrhage in seemingly worse prognosis patients.

The initial neurological status, hematoma volume, ventricular perforation are important for predicting the course of sICH, especially early mortality [58].

The highest percentage of deaths recorded in the non-statin-treated subgroup could be associated with non-adherence to statins, but it should be noted the initial significantly worse clinical status of these patients relative to subgroup Ib and the worse radiological parameters of hemorrhage in this group of patients compared to the other patients. These differences resulted from the fact that dyslipidemia, rather than baseline neurological and radiological parameters, was the only criterion for subgrouping.

At this stage of the study, it can be concluded that statins do not show negative effects on the severity of the course of sICH. We observed that both the continuation of statin treatment, included before sICH, and the use of the drug in the acute phase of the disease had no adverse effect on the depth of neurological deficit, as well as on the risk of in-hospital death. The lack of comparability of subgroup Ia and Ib patients in terms of initial clinical status and radiological characteristics of hemorrhage does not allow us to make a definite statement about the critical importance of statin use for a better course of hemorrhage.

Nevertheless, this stage of our analysis allows us to favor the concept of including statin treatment, at least with co-morbid dyslipidemia, also in the acute phase of sICH.

In the third stage, we analyzed the status of patients in the post-hospital period, up to 90 days after the onset of sICH. Patients in both subgroups and group II showed further improvement on the NIHSS scale, with the highest percentage among patients taking statins. Although improvements were observed regarding functional capacity in all analyzed patients, patients taking statins achieved particularly beneficial results (mRS close to 2, Barthel scale score of 80–83), corresponding to the ability to take care of one’s own needs in daily life independently/almost independently [59,60].

Undoubtedly, the post-hospital symptomatic treatment and rehabilitation prescribed to patients of all groups had an impact on improving the patients’ performance. We have verified information as to their neurological and functional status and the use of statins, but we have no data as to the other aspects of post-hospital therapy, including systematic rehabilitation, proper diet, and taking other medications.

In a study by Pan Y.S. et al. evaluating functional performance in 3218 Chinese patients with sICH at 3- and 12-months post-onset for in-hospital statin use, they found that statin use was associated with better functional outcome as defined by mRS 0–2 score compared to patients not using statins in the hospital. These patients had a lower 3-month mortality rate [61].

In a more recent paper, Doerrfuss et al. analyzed 919 cases with sICH using data from the Virtual International Stroke Trials Archive (VISTA) database. They evaluated the effect of statin use in the acute phase of sICH and its continued administration on functional performance outcomes. This study demonstrated that early inclusion of statins (defined as therapy administered up to 48h after the onset of sICH) and continued use of statins in the early phase of the disease are associated with beneficial functional outcome after 90 days, expressed in mRS ≤ 3 points [62]. Our observations are fully consistent with the results of the above study.

It cannot be denied that the post-hospital outcome was also influenced by the initial condition of the patients in the acute phase of the disease, which was significantly worse in patients in subgroup Ia compared to subgroup Ib, but, importantly, was not significantly different compared to group II. However, patients in this group, already taking statins in the prehospital period, significantly burdened with cardiovascular risk factors, had better results at the 90-day assessment than patients in subgroup Ia. Considering the above data, one should look for a beneficial effect of statins on the functional status of patients, also in the post-hospital period, at least up to 90 days after the onset of sICH.

Currently, a growing number of studies have shown that various mechanisms with protective effects may benefit survival and functional status in sICH, but these have still not been fully defined. Possible interactions of statins with some pathways of secondary brain damage induced by sICH have provided inspiration for further research on these drugs in this particular group of patients. The protective effect of statins on the course of sICH is believed to be due to their neuroprotective effect [37]. Nerve tissue damage caused by the forming hematoma triggers a cascade of inflammatory response and the development of brain edema [16,17].

Experimental studies which focus on the role of statins in the border zone of neural tissue and hematoma and their effects on reducing edema, inflammation, blood–brain barrier status and improving regional blood flow point to potential protective mechanisms of the drug: antiapoptotic, anti-inflammatory, antioxidant and angiogenesis-stimulating effects [18,19,20,21,22].

In human studies, despite the possibility of coexisting factors interfering with this theory, it has also been assumed that the use of statins can reduce cerebral edema [63,64,65].

The fourth stage of this study, which included survival analysis, revealed that significantly fewer patients of both subgroups I and group II died in the post-hospital period compared to the hospitalization period. Most deaths occurred by the 10th day after the illness. This was especially true for subgroup Ia and group II.

Similar observations have been made by other researchers. In an analysis of the VISTA database, they found a significant reduction in 90-day mortality among patients with early statin therapy, and a reduction, although not statistically significant, in patients continuing statin therapy compared with patients without hypolipemic therapy [62]. Tapia Perez J.H et al., in their 2015 meta-analysis, showed a significant reduction in 3-month mortality in statin-treated patients (27.3% vs. 33% in non-treated patients) [66]. A 2017 meta-analysis by Lei et al. showed that statin use during hospitalization for sICH significantly reduces mortality (the probability of death decreases by up to 60%) [67].

During this study, we noted no adverse effects of statins.

### Study Limitations

The small size of group II can be considered a weakness of this study. At the time of this study, there were more hospitalized patients with brain hemorrhage previously taking statins. However, these were patients treated with anticoagulants or the cause of the hemorrhage was secondary to coagulopathy, brain tumor, or vascular malformation. Only a few patients in group II were taking rosuvastatin in the pre-hospital period. The others were using atorvastatin. This did not allow a comparison of the use of the two statins in relation to the stated goals of this study. Consecutively enrolled group II patients were admitted to the hospital, with the vast majority of them previously taking atorvastatin 20 mg. We also included individual cases who had rosuvastatin on, given the similarity of the two statins in terms of potency (LDL fraction reduction) and half-life.

We noted the small number of patients with a previous history of sICH and using statins, which did not allow an objective analysis of the recurrence of sICH in terms of statin intake. A longer than 90-day follow-up period would be needed to assess the recurrence of sICH in terms of statin intake.

Comparability of the analyzed groups in terms of clinical and radiological prognostic factors in spontaneous intracerebral hemorrhage would have provided a stronger basis for making statements about the beneficial effect of statin use in this group of patients.

## 5. Conclusions

Regardless of the mentioned limitations of this study, the following conclusions emerge:

1. The use of statins in the pre-sICH period did not adversely affect the patients’ initial neurological status or the results of initial neuroimaging examinations.
2. Continued statin therapy prior to the onset of sICH or the inclusion of statins in acute treatment in patients with sICH and dyslipidemia does not worsen the course of the disease and the in-hospital prognosis. Statin therapy should not be discontinued during the acute phase of sICH.
3. To conclude the eventual beneficial effect on the functional performance and survival of patients after sICH onset, comparability of the analyzed groups in terms of clinical, radiological and other prognostic factors in spontaneous intracerebral hemorrhage would be needed. Future studies are needed to confirm these findings.

## Figures and Tables

**Table 1:** Risk factors for stroke in patients with sICH classified in groups I and II.

Parameter	Group I *n* = 122	Group II *n* = 31	*p* I vs. II
Age (years) Mean ± SD	68 ± 12.5	72 ± 9.1	0.0369 *
BMI Mean ± SD	26.3 ± 5.7	26.5 ± 5.5	0.9729 *
Woman n (%)	53 (44.4%)	17 (54.8%)	0.2554 **
Hypertension *n* (%)	108 (88.5%)	30 (96.8%)	0.1678 **
Ischemic heart disease *n* (%)	15 (12.3%)	14 (45.2%)	<0.001 **
Past myocardial infarction *n* (%)	8 (6.6%)	6 (19.4%)	0.0273 **
Atrial fibrillation *n* (%)	6 (4.9%)	1 (3.2%)	0.6872 **
Type 2 diabetes *n* (%)	28 (23%)	15 (48.4%)	0.0049 **
History of ischemic stroke *n* (%)	13 (10.7%)	11 (35.5%)	0.0007 **
History of intracerebral hemorrhage *n* (%)	9 (7.4%)	1 (3.2%)	0.4036 **
Dyslipidemia *n* (%)	57 (46.7%)	28 (90.3%)	<0.001 **
Cigarette smoking *n* (%)	42 (34.4%)	14 (45.1%)	0.2678 **
Alcohol consumption *n* (%)	33 (27%)	5 (16.1%)	0.2089 **
Use of antiaggregants *n* (%)	23 (18.9%)	20 (64.5%)	<0.001 **
SBP value at the time of hospital admission (mmHg) Mean ± SD	166.6 ± 30.2	163.1 ± 30	0.4635 *
DBP value at the time of hospital admission (mmHg) Mean ± SD	86.6 ± 10.4	82.1 ± 13.6	0.0014 *
Glycemia at the time of admission to the hospital (mg/dL) Mean ± SD	142.6 ± 60.7	156.2 ± 52.7	0.0809 *
TC concentration (mg/dL) Mean ± SD	180.7 ± 47.1	144.02 ± 34.1	0.0002 *
LDL concentration (mg/dL) Mean ± SD	112.8 ± 42.6	83.8 ± 33.7	0.0016 *
HDL concentration (mg/dL) Mean ± SD	55.4 ± 20.1	46.7 ± 12.9	0.0315 *
TG concentration (mg/dL) Mean ± SD	113.1 ± 48.8	105.7 ± 25.7	0.8683 *

* Mann–Whitney U test, ** Pearson’s Chi square test, mean—arithmetic mean, ±SD—standard deviation, and *n*—number of patients.

**Table 2:** Severity of intracerebral hemorrhage on clinical evaluation and neuroimaging examination on day 1 in patients with sICH classified as group I and group II.

Parameter (at the Time of Admission to the Hospital)	Group I *n* = 122	Group II *n* = 31	*p* I vs. II
NIHSS score Mean ± SD	10.9 ± 8.1	12 ± 12	0.6581 *
Volume of hemorrhagic foci on head CT scan (mm^3^) Mean ± SD	28,077.3 ± 43,844.7	25,281.5 ± 39,830	0.8855 *
Midline shift on head CT scan (mm) Mean ± SD	2.9 ± 5	2.8 ± 3	0.5859 *
Percentage of patients with brain edema on CT scan, *n* (%)	95 (78%)	22 (71%)	0.4201 **
Percentage of patients with penetration of the sICH into the ventricular system on CT scan of the brain, *n* (%)	46 (39%)	10 (33%)	0.4622 **
Percentage of patients with subarachnoid intussusception on brain CT, *n* (%)	10 (8.1%)	3 (9.8%)	0.7902 **

* Mann–Whitney U test, ** Pearson’s Chi square test, mean—arithmetic mean, ±SD—standard deviation, and *n*—number of patients.

**Table 3:** Risk factors for stroke in patients with sICH classified as subgroups Ia, Ib and II.

Parameter	Group Ia *n* = 66	Group Ib *n* = 56	Group II *n* = 31	*p* Ia vs. Ib	*p* Ia vs. II	*p* Ib vs. II
Age (years) Mean ± SD	68.5 ± 18.8	67.5 ± 10.9	73.7 ± 9.1	1.0	0.3156	0.078
Woman *n* (%) Mean ± SD	26.6 ± 6.5	26 ± 4.9	26.5 ± 5.5	1.0	1.0	1.0
Hypertension *n* (%)	29 (43.9%)	24 (42.9%)	17 (54.8%)	1.0	1.0	1.0
Ischemic heart disease *n* (%)	57 (86.4%)	51 (91%)	30 (96.8%)	1.0	1.0	1.0
Past myocardial infarction *n* (%)	9 (13.6%)	6 (10.7%)	14 (45.2%)	1.0	0.0373	0.0237
Atrial fibrillation *n* (%)	4 (6.1%)	4 (7.1%)	6 (19.4%)	1.0	0.8755	1.0
Type 2 diabetes *n* (%)	4 (6.1%)	2 (3.6%)	1 (3.2%)	1.0	1.0	1.0
History of ischemic stroke *n* (%)	18 (27.3%)	10 (17.9%)	15 (48.4%)	1.0	0.2823	0.0557
History of intracerebral hemorrhage *n* (%)	10 (15.2%)	3 (5.4%)	11 (35.5%)	1.0	0.3208	0.0605
Dyslipidemia *n* (%)	7 (10.1%)	2 (3.6%)	1 (3.2%)	1.0	1.0	1.0
Cigarette smoking *n* (%)	21 (31.8%)	21 (37.5%)	14 (45.1%)	1.0	0.8702	1.0
Alcohol consumption *n* (%)	23 (34.8%)	10 (17.9%)	5 (16.1%)	0.3192	0.4132	1.0
Use of antiaggregants *n* (%)	12 (18.2%)	11 (19.6%)	20 (64.5%)	1.0	0.0007	0.0016
SBP value at the time of hospital admission (mmHg) Mean ± SD	163 ± 32.7	170.2 ± 26.8	163.1 ± 30	0.3631	1.0	0.5394
DBP value at the time of hospital admission (mmHg) Mean ± SD	81.4 ± 10.4	83.2 ± 12.5	82.1 ± 13.6	0.5380	0.0457	0.0017
Glycemia at the time of admission to the hospital (mg/dL) Mean ± SD	155.1 ± 72.9	127.8 ± 37.4	156.2 ± 52	0.1824	1.0	0.0501
TC concentration (mg/dL) Mean ± SD	150.1 ± 26.5	209.4 ± 34.6	144.2 ± 34.1	0.0015	0.1974	<0.001
LDL concentration (mg/dL) Mean ± SD	61.2 ± 16.5	128.5 ± 41.9	83.8 ± 33.7	0.0009	0.5672	<0.001
HDL concentration (mg/dL) Mean ± SD	57.5 ± 14.4	56.1 ± 21.6	46.7 ± 12.9	1.0	0.2318	0.0680
TG concentration (mg/dL) Mean ± SD	92.3 ± 25.7	124.6 ± 52.7	105.7 ± 25.7	0.2177	1.0	1.0

Kruskal–Wallis rank-sum ANOVA test *n*-number of patients, mean—arithmetic mean, and ±SD—standard deviation.

**Table 4:** The clinical course of spontaneous intracerebral hemorrhage in the analyzed patients of subgroups Ia, Ib and group II in the in-hospital period, as well as an assessment of their functional performance and survival within 90 days of the onset of sICH.

Parameter		Group Ia	Group Ib	Group II	*p*	*p*	*p*
		*n* = 66	*n* = 56	*n* = 31	Ia vs. Ib	Ia vs. II	II vs. Ib
Head CT scan							
Volume of hemorrhagic foci [mm^3^] (mean ± SD)	1 day	43,101.1 ± 54,092.6	10,048.8 ± 111,955.5	25,281.5 ± 33,751.7	<0.001 *	0.1415 *	0.1299 *
Midline shift [mm] (mean ± SD)	1 day	4.7 ± 6.1	0.6 ± 1.4	2.8 ± 5.5	<0.001 *	0.0489 *	0.2772 *
Percentage of patients with cerebral edema [%]	1 day	83.3	71.4	71	0.7739 *	0.9806 *	1.0 *
Percentage of patients with sICH penetration into the ventricular system [%]	1 day	50	26.8	32.3	0.0822 *	0.4785 *	1.0 *
Percentage of patients with subventricular intussusception [%]	1 day	13.7	1.8	9.7	0.7804 *	1.0 *	1.0 *
Neurological examination							
NIHSS score [points] (mean ± SD)	1 day	13.8 ± 8.3	7.5 ± 6.2	12 ± 9.3	<0.001 *	0.6864 *	0.0733 *
	discharge	13.6 ± 11	6.2 ± 7.5	10.6 ± 11.3	0.0003 *	0.6291 *	0.1588 *
	90 days	5.7 ± 6.1	2.7 ± 3.4	2.2 ± 2.2	0.0499 *	0.1230 *	1.0 *
Percentage of patients with improvement on the NIHSS scale [%]	discharge	40.9	62.5	67.7	0.0201 ***	0.0121 ***	0.6232 ***
	90 days	56.3	81.1	90.1	0.0061 ***	0.0141 ***	0.1202 ***
mRS [points] (mean ± SD)	discharge	4.3 ± 1.9	3.2 ± 1.8	4.1 ± 1.7	0.0005 *	0.8578 *	0.1320 *
	90 days	3.0 ± 1.8	2.1 ± 1.5	2.4 ± 1.4	0.0275 *	0.4682 *	1.0 *
Percentage of patients with improve-ment mRS [%]	From discharge to 90 days (post-hospital period)	35.3	62.8	62	0.0161 ***	0.0542 ***	0.2301 ***
Barthel scale [Points] (mean ± SD)	discharge	43.5 ± 40.2	63.3 ± 34.4	47.7 ± 32.9	0.0184 *	1.0 *	0.3491 *
	90 days	58.3 ± 40.6	83.0 ± 26.7	80.0 ± 28.6	0.0109 *	0.2316 *	1.0 *
Percentage of patients with improvement in Barthel scale [%]	From discharge to 90 days (post-hospital period)	50	77.4	89.5	0.0262 ***	0.0051 ***	0.5331 ***
Mortality evaluation							
Mortalities [number of patients]	1 day-90 days	28	9	8	0.0042 ****	0.2228 ****	0.1371 ****
Percentage of patients who died [%]	1 day-discharge (in-hospital period)	34.8	10.7	22.6	0.0018 ***	0.2201 ***	0.1421 ***
	From discharge to 90 days (post-hospital period)	7.6	5.4	3.2	0.6202 ***	0.4103 ***	0.6522 ***
Comparison of in-hospital and post-hospital outcomes of group Ia, Ib and group II							
Parameter		Group Ia *p*		Group Ib *p*		Group II *p*	
NIHSS score [points]	1 day-discharge	0.2142 **		0.0013 **		0.0273 **	
	90 day-discharge	0.0080 *		0.000006 **		0.0001 **	
mRS [Points]	Discharge-90 day	0.0022 **		0.000006 **		0.0015 **	
Barthel scale [Points]	Discharge-90 day	0.0024 **		0.00001 **		0.0005 **	

* Kruskal–Wallis test. ** Wilcoxon paired rank order test. *** Pearson’s chi square test for 2 × 2 tables. **** Test chi square of 2 × 2 tables (Kaplan–Meier).

## Data Availability

The data cannot be made publicly available due to privacy regulations.
